# The complete chloroplast genomes of two *Taraxacum* species, *T. platycarpum* Dahlst. and *T. mongolicum* Hand.-Mazz. (Asteraceae)

**DOI:** 10.1080/23802359.2016.1176881

**Published:** 2016-06-20

**Authors:** Jin-Kyung Kim, Jee Young Park, Yun Sun Lee, Sun Min Woo, Hyun-Seung Park, Sang-Choon Lee, Jung Hwa Kang, Taek Joo Lee, Sang Hyun Sung, Tae-Jin Yang

**Affiliations:** a Department of Plant Science, Plant Genomics and Breeding Institute, and Research Institute of Agriculture and Life Sciences, College of Agriculture and Life Sciences, Seoul National University, Seoul, Republic of Korea;; b College of Pharmacy and Research Institute of Pharmaceutical Science, Seoul National University, Seoul, Republic of Korea;; c Hantaek Botanical Garden, Yongin, Gyeonggi-Do, Republic of Korea;; d Crop Biotechnology Institute/GreenBio Science and Technology, Seoul National University, Pyeongchang, Republic of Korea

**Keywords:** Chloroplast, genome sequence, *Taraxacum mongolicum*, *Taraxacum platycarpum*

## Abstract

*Taraxacum platycarpum* and *Taraxacum mongolicum* are perennial plants utilized for medicinal purposes in the family Asteraceae. The complete chloroplast genome sequences of the two species were characterized by *de novo* assembly with whole genome sequencing data. The chloroplast genomes of *T. platycarpum* and *T. mongolicum* were 151,307 and 151,451 bp in length, respectively, and showed a typical quadripartite structure. The chloroplast genomes of both species contained the same number of genes, 79 protein-coding genes, 29 tRNA genes and 4 rRNA genes. Phylogenetic analysis indicated that the two *Taraxacum* species were grouped with *T. officinale*, all of which showed sister relationship with *Lactuca sativa*.

*Taraxacum platycarpum* and *Taraxacum mongolicum* are perennial plants belonging to the family Asteraceae, the largest families of flowering plants. The genus *Taraxacum* consists of over 2500 species which is widely grown in the warm temperate areas of the Northern Hemisphere (Richards 1973). *Taraxacum* species have several curative properties including diuretic, choleretic and anti-inflammatory activities and so have long been used for traditional medicinal herbs (Schütz et al. 2006). The genus *Taraxacum* is an evolutionary and taxonomical complex taxa, because of complex hybridity and coexistence of agamosperms, which causes great difficulties in the phylogenetic study of this genus (Kirschner et al. 2003; Drábková et al. 2009). In this study, we determined the complete chloroplast genome sequences of *T. platycarpum* and *T. mongolicum*. These sequences will be valuable genetic resource for further molecular study and phylogenetic analysis of *Taraxacum* species with other species in the family Asteraceae.

*Taraxacum platycarpum* and *T. mongolicum* plants were collected from Chuncheon and Namwon, respectively, in Korea and maintained by Hantaek Botanical Garden (http://www.hantaek.co.kr), Yongin, Korea. Total genomic DNAs were isolated from fresh leaves of each species using a modified cetyltrimethylammonium bromide protocol (Allen et al. 2006). Paired-end (PE) libraries were prepared and sequenced using an Illumina MiSeq platform (Illumina, San Diego, CA) by Lab Genomics Inc. (http://www.labgenomics.co.kr), Seongnam, Korea. PE reads of 1.6 and 1.2 Gb were obtained for *T. platycarpum* and *T. mongolicum*, respectively, and each species was independently assembled by a CLC *de novo* genome assembler (v. beta 4.6, CLC Inc., Aarhus, Denmark), as mentioned in Kim et al. (2015a, b). The representative chloroplast contigs were selected, ordered and merged into a single draft sequence by comparing with the previously obtained chloroplast sequence of *T. officinale* (KU361241) as a reference (Kim et al. 2016). The draft sequences were confirmed and manually corrected by PE read mapping. The genes in the chloroplast genomes were annotated using the DOGMA program (Wyman et al. 2004) and BLAST searches.

The complete chloroplast genomes of *T. platycarpum* (GenBank accession KU736960) and *T. mongolicum* (GenBank accession KU736961) were 151,307 and 151,451 bp in length, respectively. As a typical quadripartite structure found in chloroplast genomes of other angiosperms, the genomes of *T. platycarpum* and *T. mongolicum* were separated into four distinct parts, a large single copy (LSC) region (83,922 and 84,052 bp), a short single copy (SSC) region (18,507 and 18,541 bp) and a pair of inverted repeats (IRa and IRb) regions (24,439 and 24,429 bp). A total of 112 genes including 79 protein-coding genes, 29 tRNA genes and 4 rRNA genes were identified in the both chloroplast genomes. The sequence homology of complete chloroplast genomes between *T. platycarpum* and *T. mongolicum* was 99.1%.

Phylogenetic analysis with other 15 species in the family Asteraceae was carried out based on common 68 chloroplast protein coding sequences, using a maximum likelihood analysis of MEGA 6.0 (Tamura et al. 2013) with 1000 bootstrap replicates. The phylogenetic tree showed that *T. platycarpum* and *T. mongolicum* were grouped with *T. officinale*, another *Taraxacum* species, all of which showed sister relationship with *Lactuca sativa* (lettuce, a leaf vegetable) in the Cichorioideae subfamily, as expected (Figure 1).

**Figure 1.**
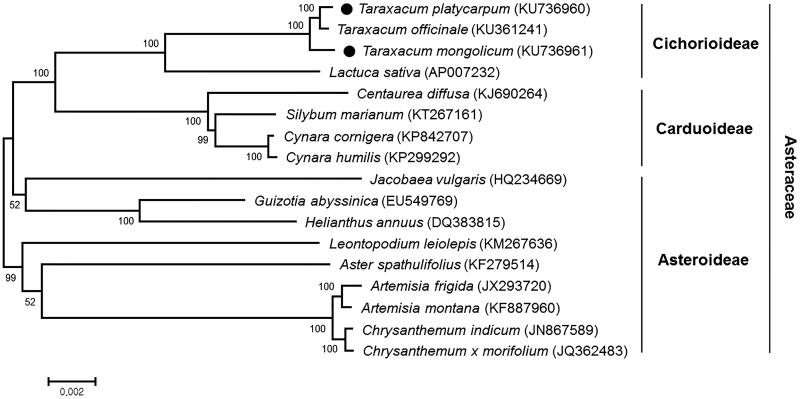
Maximum likelihood phylogenetic tree of *T. platycarpum* and *T. mongolicum* with related 15 species in the family Asteraceae based on common 68 chloroplast protein-coding genes. Numbers in the nodes are the bootstrap values from 1000 replicates.
